# High-throughput sequencing reveals omnivorous and preferential diets of the rotifer *Polyarthra in situ*

**DOI:** 10.3389/fmicb.2022.1048619

**Published:** 2022-12-21

**Authors:** Diwen Liang, Hailin Luo, Chunrong Huang, Zhen Ye, Shuangshuang Sun, Jiahua Dong, Mingyi Liang, Senjie Lin, Yufeng Yang

**Affiliations:** ^1^State Environmental Protection Key Laboratory of Urban Ecological Simulation and Protection, South China Institute of Environmental Sciences, MEE, Guangzhou, China; ^2^Institute of Hydrobiology and Research Center of Low Carbon Economy for Guangzhou Region, Jinan University, Southern Marine Science and Engineering Guangdong Laboratory (Zhuhai), Guangzhou, China; ^3^Department of Marine Sciences, University of Connecticut, Groton, CT, United States

**Keywords:** zooplankton, 18S rDNA, food web, feeding response, food spectrum, dietary analysis

## Abstract

Knowledge of *in situ* diet of widespread rotifers is crucial for accurately understanding the trophic position, ecological function, and adaptability to environmental changes in aquatic ecosystems. However, it is challenging to achieve the *in situ* diet information due to the lack of efficient and comprehensive methods. Here, we investigated the diet composition of *Polyarthra* in a subtropical lake using high-throughput sequencing (HTS) of a rRNA metabarcode for *Polyarthra* and ambient water samples. After eliminating *Polyarthra* sequences, a total of 159 operational taxonomic units (OTUs) from taxa in 15 phyla were detected from *Polyarthra* gut content samples. Most of the OTUs belong to Chlorophyta, followed by unclassified Fungi, Chrysophyta, Dinoflagellata, Ciliophora, Bacillariophyta, Cryptophyta, Arthropoda, Cercozoa, Mollusca, Apicomplexa, Haptophyta, Amoebozoa, Chordata and other eukaryotes. Our results showed that *Polyarthra* mainly grazed on Chlorophyta, which may result from the high relative abundance of Chlorophyta in ambient waters. In contrast, Chrysophyceae and Synurophyceae were enriched in *Polyarthra’s* gut, indicating that this rotifer prefers these taxa as food. Moreover, correlation analysis showed that total nitrogen, transparency, depth, Chlorophyll-*a* and total phosphorus were key factors for the variation of the eukaryotic community in the *Polyarthra* gut contents. When the concentration of nutrients in the water environment decreased, *Polyarthra* shifted from herbivorous feeding to more carnivorous feeding. Thus, *Polyarthra* is generally omnivorous but preference for Chrysophytes and Synurophytes, and it responds to the environmental changes by adopting a flexible feeding strategy. This could partly explain why the widespread rotifers have apparently wide tolerance toward spatial and environmental changes.

## Introduction

Rotifers, a group of essential zooplankton in aquatic ecosystems, are sensitive to changes of the environment, acting as reliable indicators of the trophic status (Sládeček, 1983; Liang et al., 2020). Rotifers connect primary producers and secondary consumers, playing an important role not only in the food chain but also the micro-food web (Arndt, 1993; Galir Balkić, 2019). *Polyarthra dolichoptera* and *Polyarthra vulgaris*, cosmopolitan rotifer species, are more tolerant to seasonal changes than other rotifers and widely dominant not only in freshwaters but also brackish waters (Liang et al., 2022). However, it is still unclear why these rotifers have a strong adaptability to environmental changes. On the one hand, their higher genetic diversity may be the reason for them to adapt to specific habitats (Obertegger et al., 2014). On the other hand, the adaptation for survival of widespread species could be attributed to their tolerances to harsh environment (e.g., thermal and toxins stress), as well as their wide food spectrum (Boersma et al., 2016; Liang et al., 2017; Lin et al., 2018).

Studying the diets of widespread species is an important approach to clarify their trophic position and ecological function in aquatic ecosystems, understanding exchanges of material and energy transfer through food webs affected by environmental changes (Boersma et al., 2016; Hu et al., 2018). The diets of primary consumers may reflect both environmental quality and their survival status. Therefore, the variations of diet composition may help us to understand their survival strategies. Many studies demonstrated that *Polyarthra* not only grazed on phytoplankton but also consumed ciliates (Arndt, 1993; Joaquim-Justo et al., 2004). However, its feeding preference among different prey species still needs further investigation.

Previous dietary studies on zooplankton have mainly been based on traditional methods, such as morphological identification on gut contents, feeding behavioral observation under microscope and laboratory experiments (Arndt, 1993; Drzewicki et al., 2015; Sarma et al., 2019). These approaches do not provide complete information on food spectrum of rotifers in natural waters. Although natural dietary information can be obtained from feeding behavioral observation of wild-caught zooplankton, the currently available microtechnique is challenging because some zooplanktons with strong escaping ability are hard to follow and their fragmental prey can be extremely difficult to identify (Hu et al., 2014). While information from incubation experiments has constituted the backbone of aquatic planktonic trophic ecology, it falls short in documenting the full range of the trophic linkages among the complex components in the natural environment (Hu et al., 2015). In addition, pigment analysis has been used to study the prey diversity, but it is limited to phytoplankton and are of low taxonomic resolution (Turner, 2004). The approach using fluorescent microparticles successfully estimated the predation of rotifers on ciliates but only under semi-*in situ* conditions (Joaquim-Justo et al., 2004). Also, stable isotope analysis of fatty acids is reliable in tracing sources of nitrogen or carbon and provide information of trophic level, but still could not depict the whole picture of diet composition (Gonçalves et al., 2012; Boersma et al., 2016).

In recent years, DNA-based methods have been widely applied to study the feeding ecology of microzooplankton due to its sensitivity, specificity and rapidness over traditional methods. The 18S ribosomal RNA gene (18S rDNA) is widely used in PCR as a reliable taxonomic marker because it consists of multiple copies in the genomes of eukaryotic organisms and the hypervariable region can be used to distinguish genetic differences among species (Hu et al., 2014). Universal 18S rDNA primers (targeting the hypervariable V4 region) have been proven as an effective and efficient method in studying the eukaryotic diversity (Cheung et al., 2010). Although clone-based sequencing has successfully explored the *in situ* diet of copepod, the data generated by this approach only provide general information on the structure of eukaryote communities but are not sufficient for meaningful comparisons among libraries (Cheung et al., 2010). On the contrary, high-throughput sequencing (HTS) can provide semi-quantitative information on the contribution of different prey to the diet of a consumer and detect rare prey species in gut contents (Ho et al., 2017). A weak but positive relationship between relative read abundance (eDNA) and biomass (traditional approach) has been found in recent studies of zooplankton (Ershova et al., 2021), benthic invertebrate (Klunder et al., 2022) and fish (Stoeckle et al., 2021). HTS has been successfully used to detect the food composition of invertebrates, mollusca, fish and marine mammal (Ho et al., 2017; Lin et al., 2018). Thus, it is a promising method for dietary studies of microorganisms such as rotifers.

The aims of this study were: (1) to provide more detailed information on the diet spectrum of rotifer *Polyarthra*, exploring the food resources of this widespread rotifer; (2) to characterize the *in situ* diet composition of *Polyarthra* and the feeding responses to environmental changes; (3) to understand the feeding strategy that enables widespread rotifers to survive and thrive under different trophic conditions.

## Materials and methods

### Sample collection

A total of 24 samples, including 12 rotifer samples and 12 ambient water samples were collected from Lake Liuye, Changde, China (Supplementary Figure S1; Supplementary Table S1). The average interval between sampling sites was set to about four kilometers. Samples were taken on a boat at every site quarterly from December 2017 to September 2018. The ambient water samples (1 L) were collected with polyethylene bottles from the surface and subsurface layer. The water samples were filtered immediately on a 0.2 μm polycarbonate membrane (EMD Millipore GTPP04700, USA) and stored at −80°C. Rotifer samples were collected by towing a plankton net (mesh size 30 μm) horizontally at surface and subsurface depths for 5 min. To prevent changes in rotifer gut contents in the sampling process, rotifers samples were fixed on site immediately in neutral Lugol’s solution at 2% final concentration and transported in a cooler before storing at −20°C.

Water temperature (Temp), dissolved oxygen (DO), pH, and salinity, were measured using a calibrated multiprobe (YSI-Plus, USA). Water depth (Dep) was measured using a fathometer (SM-5, USA). Water transparency (SD) was measured with a Secchi disc. Chlorophyll-*a* (Chl-*a*), chemical oxygen demand (COD_Mn_), total phosphorus (TP), ammonium nitrogen (NH_4_-N) and total nitrogen (TN) were determined in the laboratory following standard analytical methods (MEE, 2002).

In the laboratory, *Polyathra dolichoptera* and *P. vulgaris* were identified and 50–100 individuals of similar size were isolated with sterilized micropipettes and Sedgewick-Rafter chambers under the stereo microscope (Supplementary Figure S2). The isolated individuals were serially rinsed four times thoroughly with double distilled water under the stereo microscope, examined to ensure no attachment of other visible organisms on the body surface and appendages. All washed individuals were transferred into a 1.5 ml Eppendorf tube for DNA extraction.

### DNA extraction, amplification, and sequencing

The *Polyarthra* rotifers in the 1.5 ml EP tube were homogenized using a sterilized disposable grinding rod. Total DNA in each rotifer sample was then extracted following the HotSHOT protocol (Montero-Pau et al., 2008). Total DNA in each ambient water sample was extracted from the polycarbonate membrane using DNA Lysis buffer + protein K + CTAB + Clean & Concentrator kit (Zymo Research, USA) methods following the improved protocol (Yuan et al., 2015). The concentrations and purities of DNA were measured on a NanoDrop 2000 Spectrophotometer.

The V4 region of the 18S rRNA gene was amplified to detect the eukaryotic communities in both *Polyarthra* gut contents and ambient waters using universal primers 528F (5′-GCGGTAATTCCAGCTCCAA-3′) and 706R (5′-AATCCRAGAATTTCACCTCT-3; Cheung et al., 2010). Thermal cycling conditions of amplification were: 3 min of denaturation at 94°C, followed by 27 cycles of 30 s at 94°C, 30 s at 55°C, 30 s at 72°C and a final extension at 72°C for 5 min. PCR reactions were performed in triplicate 20 μl mixture containing 4 μl of 5 × FastPfu Buffer, 2 μl of 2.5 mM dNTPs, 0.8 μl of each primer (5 mM), 0.4 μl of FastPfu Polymerase and 10 ng of template DNA. The PCR products were extracted from a 2% agarose gel and further purified using the AxyPrep DNA Gel Extraction Kit (Axygen Biosciences, Union City, CA, USA) and quantified using QuantiFluorTM-ST (Promega, USA) according to the manufacturer’s protocol. Purified amplicons were pooled in equimolar and paired-end sequenced on an Illumina Miseq platform (Illumina, San Diego, USA) using NEXTFLEX Rapid DNA-Seq Kit. Sequencing was conducted in the Shanghai Meiji Sequencing Centre.

### Sequencing data processing and statistical analyses

Raw FASTQ files were demultiplexed, quality-filtered by fastp and merged by FLASH to minimize the effects of random sequencing errors (Magoč and Salzberg, 2011). Briefly, sequences were removed if: (1) they contained ambiguous base calls, (2) their lengths were shorter than 240 bps or the average quality score were <20. After sequence screening, operational taxonomic units (OTUs) were clustered with a 97% similarity cutoff using UPARSE (version 7.1^1^) and chimeric sequences were identified and removed using UCHIME (Edgar, 2013). The taxonomy of each OTU representative sequence was analyzed using RDP Classifier algorithm^2^ against the NCBI nucleotide sequence database NT (NT_v20200327/18S_eukaryota) using confidence threshold of 0.7. Finally, the corresponding species information of each OUT was obtained.

Since this study was aimed at analyzing the composition of eukaryotes in the *Polyarthra* gut contents, it was necessary to remove the sequences of *Polyarthra* themselves from the samples before analysis. *Polyarthra* reads from its gut contents samples were subtracted before relative abundance (based on reads abundance) calculation. Rarefaction curves were plotted on OUT level by using Mothur for each sample (Schloss et al., 2009). Venn diagrams were generated with online tools to show the common and unique species in ambient water and *Polyarthra* samples.^3^

To calculate the differences of the eukaryotic communities between the ambient water and *Polyarthra* samples, the dissimilarity tests between groups were performed by the analysis of similarities (ANOSIM). In general, *R* > 0.75 means large difference; 0.75 > *R* > 0.5 means medium difference, 0.5 > *R* > 0.25 means small difference. Food selection of *Polyarthra* was estimated using the selectivity index *E* (Ivlev, 1961; Cotonnec et al., 2001), given by *E = (ri – pi)/(ri + pi)*. *ri* is the relative abundance of one eukaryotic genus in the diet of *Polyarthra* sample. While *pi* is the relative abundance of the same eukaryotic genus in the ambient water sample. In general, *E* > + 0.25 indicates a preference; *E* < − 0.25 indicates discrimination against particular prey items; − 0.25 < *E* < + 0.25 indicates non-selective feeding.

A heatmap was also generated using the R ‘vegan’ package. Canonical correspondence analysis (CCA) or Redundancy analysis (RDA) was used to identify the effects of environmental factors on the *in situ* diet composition of *Polyarthra*. Whether CCA or RDA model was used was determined based on the community composition by Detrended Correspondence Analysis (DCA). If the longest gradient was >4, the unimodal method (CCA) was applied. If that value was <3, the linear method (RDA) was a better choice. All the above analyses were conducted with R (version 4.1.1^4^) using ‘vegan’, ‘graphics’, ‘maptools’ and ‘ggplot2’ packages. The relative abundance difference and the significance level of species between ambient water and *Polyarthra* samples were compared by Wilcoxon ranksum test using ‘stats’ packages. The calculated value of p was gone through FDR Correction, taking FDR ≤ 0.05 as a threshold.

A phylogenetic tree was constructed based on sequences of dominant OTUs in *Polyarthra* samples using a Maximum Likelihood method with a bootstrap value of 1,000 displayed by MEGA X and the plot was generated on iTOL (Letunic and Bork, 2011).

## Results

### Compositions of eukaryotes in the *Polyarthra* gut contents and the ambient waters

**Figure 1:**
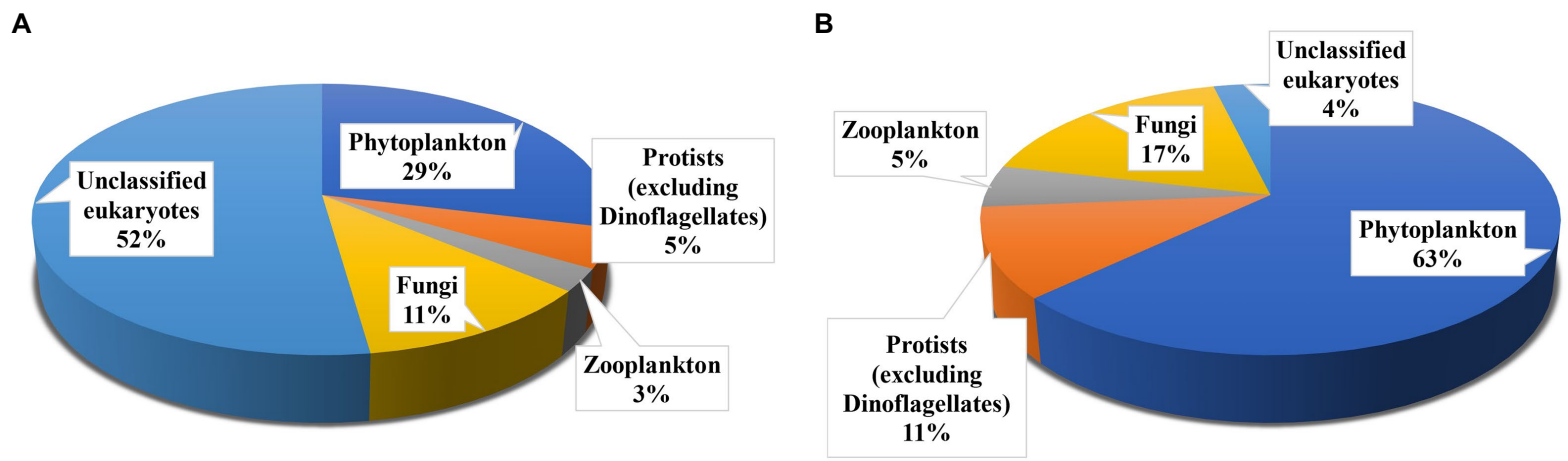
Operational taxonomic unit (OTU) richness of main groups in the ambient waters **(A)**, and *Polyarthra* gut contents samples **(B)**.

Rarefaction curves for all the samples were nearly saturated (Supplementary Figure S3), suggesting sufficient sequencing depth for this study. High-throughput sequencing of 18S rRNA V4 region yielded 715,818 clean metabarcode sequences from all 12 ambient water samples. After blasting against NCBI using BLASTN, a total of 1,348 OTUs in ambient water samples were divided into five groups. The highest numbers of OTUs was observed as unclassified eukaryotes (704 OTUs), followed by phytoplankton (including dinoflagellates; 389 OTUs), fungi (156 OTUs), protists (excluding dinoflagellates; 63 OTUs), and zooplankton (36 OTUs; Figure 1A).

After eliminating *Polyarthra* sequences, 6,789 sequences were obtained for all 12 *Polyarthra* gut contents samples. A total of 159 OTUs were observed in *Polyarthra* gut contents. Most of the observed OTUs belonged to phytoplankton (100 OTUs), followed by fungi (28 OTUs), protozoa (17 OTUs), zooplankton (8 OTUs) and unclassified eukaryotes (6 OTUs; Figure 1B). Moreover, 129 OTUs were shared between the ambient waters and the *Polyarthra* gut contents samples (Supplementary Figure S4).

Phylogenetic tree of OTUs from *Polyarthra* gut contents samples based on Maximum Likelihood showed that the 159 OTUs sequences could be classified into 15 Phyla and 39 Classes (Figure 1B). A large number of phytoplankton taxa (100 OTUs) was detected in *Polyarthra* gut contents samples. Most of the observed OTUs belonged to Chlorophyta (54 OTUs), followed by unclassified Fungi (28 OTUs), Chrysophyta (17 OTUs), Dinoflagellata (10 OTUs), Ciliophora (10 OTUs). Bacillariophyta (9 OTUs), Cryptophyta (9 OTUs), Arthropoda (8 OTUs), Cercozoa (5 OTUs), Mollusca (3 OTUs), Apicomplexa (1 OTUs), Haptophyta (1 OTUs), Amoebozoa (1 OTUs), Chordata (1 OTUs) and other eukaryotes (2 OTUs).

**Figure 2:**
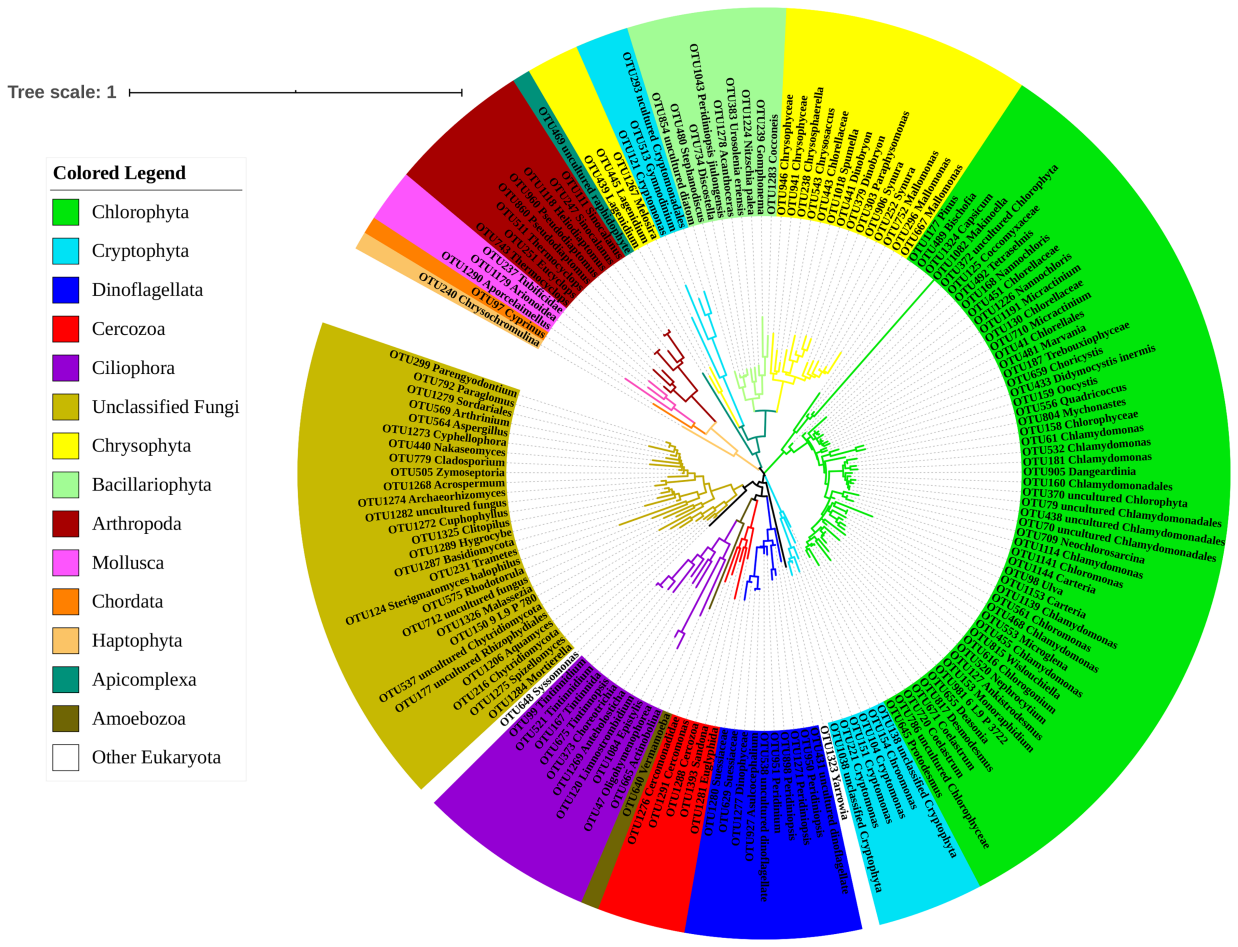
Phylogenetic tree of OTUs from the samples of *Polyarthra* gut contents based on Maximum Likelihood. The color coding of OTUs represents different phyla.

A small number of sequences of animal and macroalgal were also detected, including fishes of *Cyprinus*, molluscs of Tubificidae, Arionoidea, large zooplankton of *Sinocalanus*, *Pseudodiaptomus*, *Eucyclops*, *Thermocyclops*, and algae from the genus *Ulva* (Figure 2).

**Figure 3:**
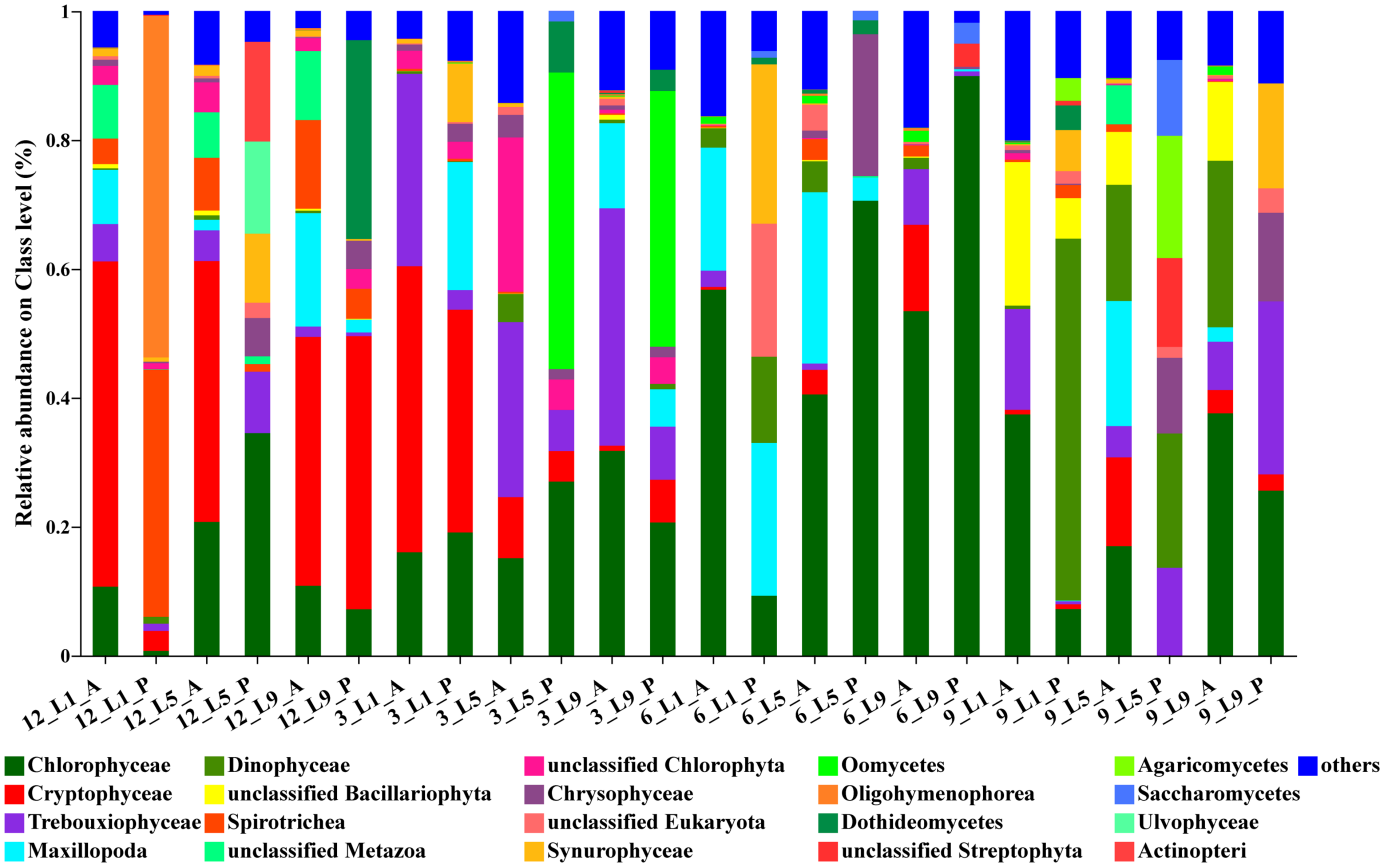
Relative abundance (based on reads abundance) of the ambient water samples and the *Polyarthra* samples on class level (top 20). *Polyarthra* reads from the 12 gut contents samples were removed before calculation. A: the ambient water samples; P: the *Polyarthra* gut contents samples; 12: December; 3: March; 6: June; 9: September.

For ambient water samples, the most abundant class was Chlorophyceae, with the average relative abundance of 28.1%. The next Class was Cryptophyceae, with an average relative abundance of 19.8%, followed by Trebouxiophyceae (10.8%), Maxillopoda (9.1%), Dinophyceae (5.4%) and Bacillariophyceae (4.1%). For *Polyarthra* gut contents samples, the most abundant class was also Chlorophyceae, with the average relative abundance of 16%. The next Class was Oligohymenophorea, with an average relative abundance of 14.6%. In addition, Dinophyceae (11.8%), Spirotrichea (11.4%), Cryptophyceae (10.2%) were also abundant (Figure 3).

The seasonal variations of eukaryotic composition of water environment at the three sites were consistent (Figure 3). Cryptophyceae dominated in the ambient water in December 2017, with the relative abundances reaching 39%–51%. The relative abundances of Trebouxiophyceae (27%–37%) increased in March 2018. While in June, Chlorophyceae dominated in the ambient water, with the relative abundances reaching 41%–57%. In September, the relative abundances of Chlorophyceae decreased to 17%–38%, and those of Bacillariophyceae increased to 8%–22%. The composition of eukaryotic phytoplankton community determined by metabarcoding was consistent with the results based on the morphological approach (Supplementary Figure S5).

The average relative abundance of Chlorophyta was the highest in the *Polyarthra* gut content samples, reaching 21.8%. However, the variations of eukaryotic composition of the *Polyarthra* gut contents were different among the three sites. In general, the relative abundance of Cryptophyceae and Oomycetes in the *Polyarthra* gut contents in December and March were greater than those in June and September. While Chlorophyceae dominated the *Polyarthra* gut contents in all seasons, and the relative abundance of Dinophyceae, Trebouxiophyceae and Chrysophyceae in the *Polyarthra* gut contents increased in September (Figure 3).

### Comparison of eukaryotic communities between the ambient waters and the *Polyarthra* gut contents samples

**Figure 4:**
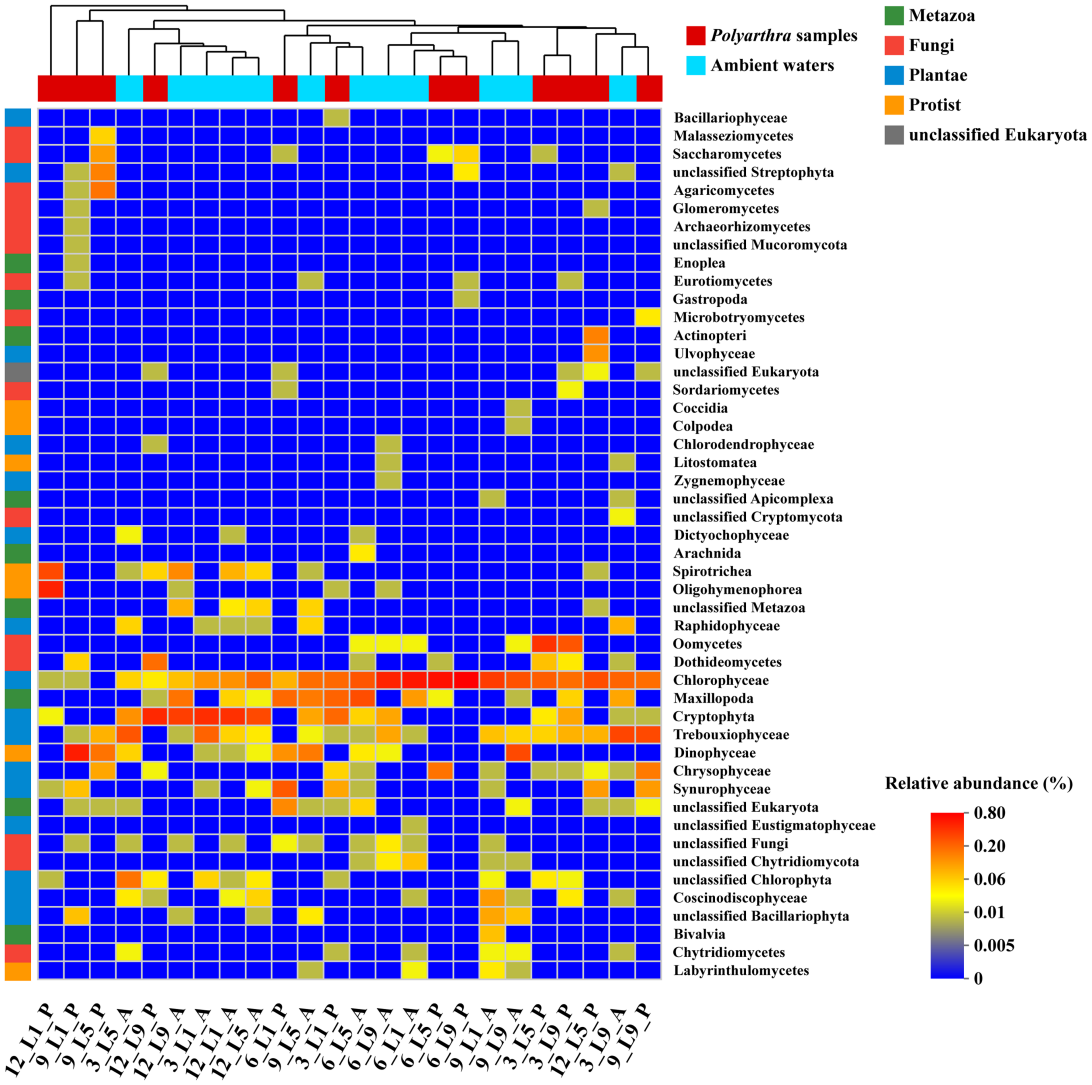
Heatmap analysis of eukaryotic communities between ambient water (A) and *Polyarthra* (P) based on Bray - Curtis distance (Top 50 abundant OTUs on Class level). Samples and taxa were automatically organized by hierarchical clustering. A: the ambient water samples; P: the *Polyarthra* gut contents samples; 12: December; 3: March; 6: June; 9: September.

The results of the community similarity analysis (ANOSIM) on the class level showed that there was small difference of eukaryotic communities between the ambient waters and the *Polyarthra* gut contents (*R* = 0.35, *p* < 0.01). The samples collected from hot season (Jun and Sep) mostly stayed together, while the ambient water samples and the *Polyarthra* gut contents samples were mostly together (Figure 4). Moreover, phytoplankton such as Chlorophyceae, Trebouxiophyceae, Crytophyta, and Dinophyceae dominated not only in the ambient waters but also in the *Polyarthra* gut contents. These results indicated that the eukaryotic community in the *Polyarthra* gut contents varied with the plankton community in the ambient waters.

**Figure 5:**
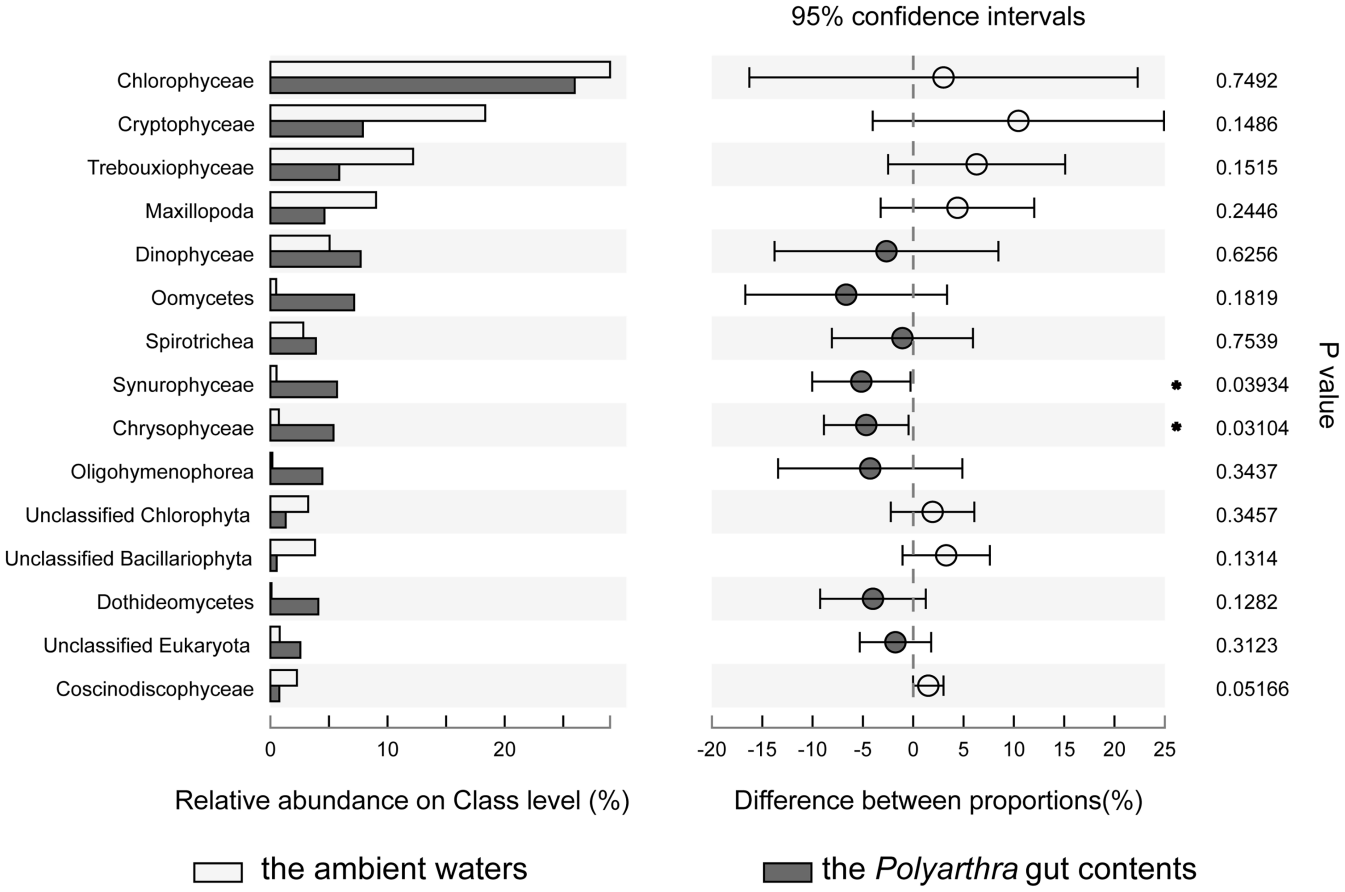
Comparison of the differences in the average relative abundance of eukaryotes on class level (top 15) between the *Polyarthra* gut contents and the ambient water samples (*p < 0.05; Wilcoxon rank-sum test).

The Wilcoxon rank-sum test was used to analyze the significant difference of species composition between the *Polyarthra* gut contents and the ambient water samples (Figure 5). Chlorophyceae, Cryptophyceae, Trebouxiophyceae, Maxillopoda, Dinophyceae dominated in both the ambient waters and the *Polyarthra* gut contents. The higher relative abundances of Dinophyceae, Oomycetes, Spirotrichea, Oligohymenophorea, Chrysophyceae and Synurophyceae were found in the *Polyarthra* gut contents than in the ambient water samples. In addition, the relative abundances of Chrysophyceae and Synurophyceae in the samples of the *Polyarthra* gut contents were significantly greater than those in ambient waters (*p* < 0.05; Figure 5).

The *E* values (Table 1) and the significant differences of eukaryotic compositions between the *Polyarthra* gut contents and the ambient water samples (Figure 5) indicated a selective feeding on Chrysophyceae and Synurophyceae. Whereas the Chrysophyceae and Synurophyceae were in relatively low concentrations in ambient waters, they were enriched in *Polyarthra* diets. By contrast with Chrysophyceae and Synurophyceae, the selectivity index showed a non-selective feeding on Chlorophyceae, Cryptophyceae and Trebouxiophyceae for *Polyarthra*, as they were dominant phytoplankton in both the *Polyarthra* gut contents and the ambient waters (Figure 5; Table 1).

**Table 1:** The food selectivity index (*E*) of *Polyarthra* on eukaryotes on class level (top 20).

Taxa on class level	The dominance index (*Y*)	The food selectivity index (*E*)			
		December	March	June	September
Chlorophyceae	0.268	0.00	0.03	0.06	−0.47
Cryptophyceae	0.156	−0.48	−0.09	−1.00	−0.69
Trebouxiophyceae	0.098	−0.04	−0.68	−0.89	0.19
Maxillopoda	0.064	−0.87	**0.32**	−0.25	−0.99
Dinophyceae	0.041	−0.09	−0.70	0.17	**0.27**
unclassified Bacillariophyta	0.023	−0.85	−1.00	−1.00	−0.74
Spirotrichea	0.023	**0.26**	−0.40	−1.00	0.10
unclassified Chlorophyta	0.022	−0.41	−0.41	−1.00	−1.00
Coscinodiscophyceae	0.017	−0.82	−0.12	−1.00	−0.35
unclassified Chytridiomycota	0.011	**0.43**	**0.61**	−1.00	−1.00
unclassified Metazoa	0.010	−0.91	0	**0.94**	−1.00
Raphidophyceae	0.010	−0.72	−1.00	−1.00	−1.00
Chrysophyceae	0.007	**0.72**	0.08	**0.90**	**0.96**
unclassified Fungi	0.006	−0.17	−0.47	−0.44	−0.13
unclassified Eukaryota	0.005	**0.39**	−0.81	**0.64**	**0.68**
Synurophyceae	0.005	**0.53**	**0.71**	**0.98**	**0.92**
Chytridiomycetes	0.003	−1.00	−0.55	−1.00	0
Oomycetes	0.003	−1.00	**0.99**	−1.00	−1.00
Oligohymenophorea	0.002	**0.97**	−0.03	−1.00	−1.00
Labyrinthulomycetes	0.001	0	0	−1.00	−1.00

Bold, indicates a preference.

### Relationships between the eukaryotic communities and environmental variables

**Figure 6:**
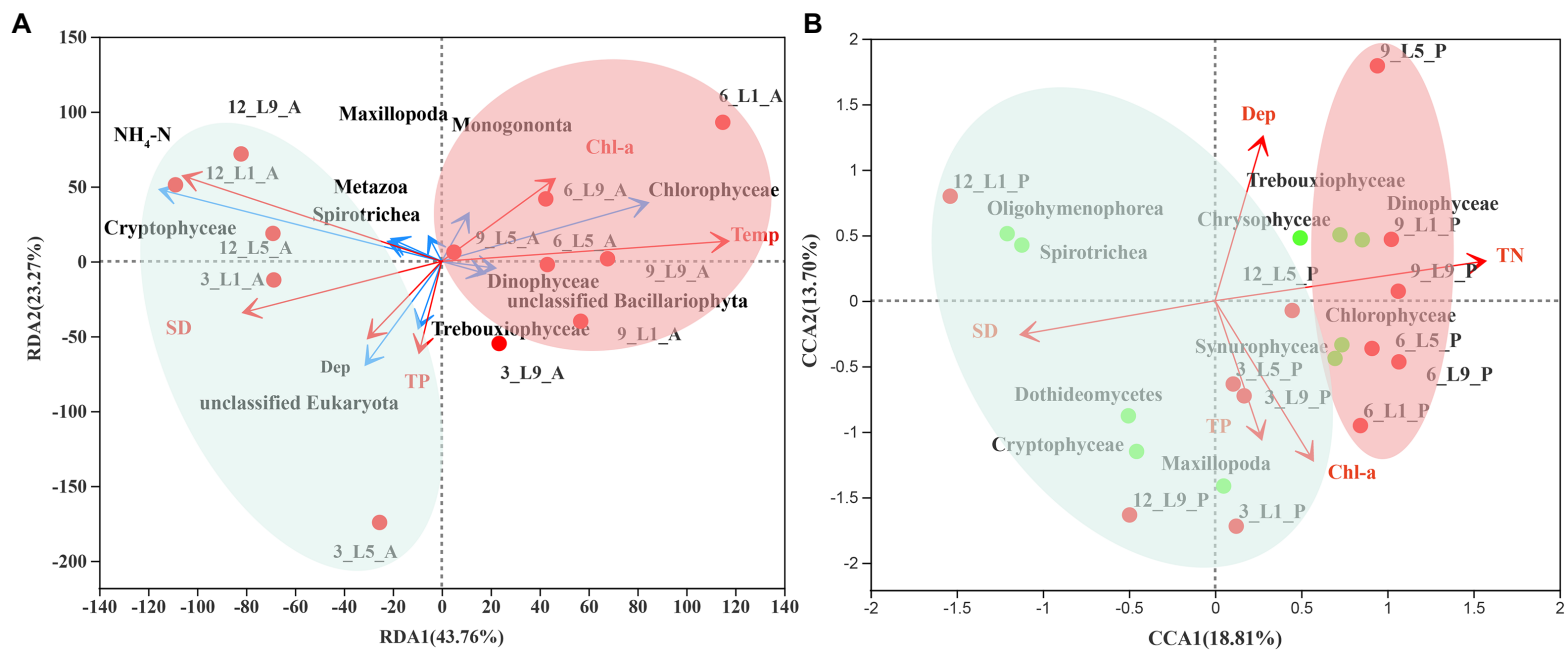
Restrictive ordination analysis of relationship between eukaryotic communities and environmental factors. **(A)** RDA of ambient water samples: Relative abundance of top 10 eukaryotes (blue arrows), environmental variables (red arrows). **(B)** CCA of *Polyarthra* samples: Relative abundance of top 10 eukaryotes. 12 and 3: December and March (cyan circle); 6 and 9: June and September (red circle).

As the longest gradient of DCA for the ambient water samples was 1.6, a RDA model was used to estimate the relationship between eukaryotic communities of the ambient waters and the environmental factors. Since the longest gradient of DCA for *Polyarthra* samples was 4.4, CCA was a better choice. For RDA, the first two ordinate axes explained 67% of the eukaryotes-environment variability in the ordination of environmental variables (Figure 6A; Supplementary Table 2). After forward selection with Monte Carlo permutation tests, only temperature, NH_4_-N, transparency, Chlorophyll-*a*, depth and total phosphorus were significant contributors to the variation of the eukaryotic community in the ambient waters.

For CCA, the first two ordinate axes explained 33% of the eukaryotes-environment variability in the ordination of environmental variables (Figure 6B; Supplementary Table S3). After forward selection with Monte Carlo permutation tests, only total nitrogen, transparency, depth, Chlorophyll-*a* and total phosphorus were significant contributors to the variation of the eukaryotic community in the *Polyarthra* gut contents.

## Discussion

### Prey diversity

A total of 159 OTUs of eukaryotes belonging to phytoplankton, metazoans, protozoans, fungi and other unclassified eukaryotes were observed in *Polyarthra* gut contents by HTS. The *in situ* diets confirmed the omnivorous feeding of *Polyarthra*, in accordance with previous laboratory experiments (Arndt, 1993; Joaquim-Justo et al., 2004; Espinosa-Rodríguez et al., 2021). Arndt (1993) summarized rotifers diets using traditional observation and found that phytoplankton (e.g., Chlorophyta, Cryptophyta), protozoa (e.g., Ciliates, Flagellates, Amoebas, Rhizopos), fungi (e.g., yeasts), bacteria (e.g., Aerobacter) and organic detritus were food resources for rotifers. Laboratory investigations showed that *Polyarthra* mainly feed on edible algae, especially *Chlamydomonas* (Gilbert and Bogdan, 1981; Wen et al., 2011). Also, it took advantage of autotrophic and heterotrophic flagellates such as *Chilomonas*, *Euglena*, *Bodo*, and *Cyathomonas* (Buikema et al., 1977; Pourriot, 1977). In the present study, 11 OTUs of different *Chlamydomonas* and 17 OTUs of protozoa (e.g., Vermamoeba, Tintinnina, Raphidophyte) were detected by HTS in the gut content of *Polyarthra*. This molecular evidence supported the notion that protozoa are important food sources for rotifers. This implies that rotifers are not only micrograzers but also predators of micro-food web and that, as proposed in the 20^th^-century (Arndt, 1993).

In the view of functional groups, *Polyarthra* is considered as raptorial and macrofilter-feeder, since it shows active grasping and piercing actions to catch single food items (Obertegger et al., 2011). However, there are few reports about rotifers feeding on crustaceans, mollusks, macrophytes and so on. Many unexpected prey taxa that were overlooked by microscopic observation could be detected from gut contents through HTS (Lin et al., 2018). A small number of sequences of fishes as *Cyprinus*, molluscs as Tubificidae, Arionoidea, arthropods as *Sinocalanus*, *Pseudodiaptomus*, *Eucyclops*, *Thermocyclops*, macroalgaes as *Ulva* were detected in the *Polyarthra* gut contents in the present study. It was also reported that copepods, the macrozooplankton, feed on metazoans that larger than itself such as Hydrozoans, Ctenophores, Arrow worms, Echinoderms, and Tunicates in the form of detritus and eggs (Ho et al., 2017; Yi et al., 2017; Xu et al., 2020). Since *Polyarthra* is a micro-zooplankton with the body length ranges from 90 to 245 μm, it is more likely to feed on the eggs and gametes than adults of arthropods, fishes and macroalgae. Besides the form of eggs or gametes, metazoans could also be ingested in the form of organic particles/detritus (Yi et al., 2017). The energy supplied by a metazoan diet with high levels of lipids and proteins, may be more accessible and easily utilized by zooplankton than plant food items (Hu et al., 2018). Additionally, *Polyarthra* usually shows significantly positive correlation with COD_Mn_ and thrives in habitats with higher nutrient content and lower transparency, supporting that it inclines to consume detritus particles (Ji et al., 2013; Liang et al., 2020).

Macroalgae is one of the potential food sources for *Polyarthra*. Microscopic observation and molecular analysis have shown that heterotrophic ciliates can acquire *Ulva* sequence by ingesting their gametes, chloroplasts and detritus (McManus et al., 2004). Also, copepods could use plant detritus as supplementary food sources when phytoplankton production is limited (Hu et al., 2015). It is possible that *Polyarthra* could take advantage of the gametes or detritus of macroalgae by adopting an “opportunistic feeding.”

Twenty eight OTUs of fungi, belonging to Chytridiomycota, Ascomycota, Basidiomycota and Mucoromycota were detected in the *Polyarthra* samples. Fungi were also found in the diet of European eel larvae and in the diet of calanoid copepods through molecular approach (Riemann et al., 2010; Xu et al., 2020). As an important component of microorganisms in aquatic ecosystems, most fungi are either parasitic or symbiotic to microorganisms including zooplankton, or attach to nutrient-rich sediments. Only a minority are free-living (Richards et al., 2012). So far, the relationship between fungi and zooplankton is still unclear (Xu et al., 2020). Thus, the fungi detected in the *Polyarthra* samples may be either parasites or constituted the diet of *Polyarthra*. Further studies combining scanning electron microscopy and fluorescence tracing are needed to confirm the trophic relationship between fungi and *Polyarthra.* In any case, this study documented the dietary information of natural rotifer *Polyarthra* and expanded the knowledge of their *in situ* food resources in detail.

### Prey preference

Our results showed that *Polyarthra* had a high ingestion on Chlorophyta in this river–lake ecosystem. It seems that Chlorophyceae is the staple diet for rotifers. *Chlorella*, *Scenedesmus* and *Chlamydomonas* have been considered the most suitable food for the laboratory cultured rotifers (Gilbert and Bogdan, 1981; Geng and Xie, 2008; Sun et al., 2020). However, the *E* values and the differences of average relative abundances in the present study suggests that *Polyarthra* mainly grazed on Chlorophyta, which may result from its “opportunistic feeding” strategy. As Chlorophyceae, Cryptophyceae, Trebouxiophyceae, Dinophyceae dominated in the ambient waters, they became more readily accessible to *Polyarthra* than other food items. On the contrary, *Polyarthra* exhibited a high preference for Chrysophyceae and Synurophyceae.

The prey preference for macro-zooplankton could be related to the nutritional state of the food items (Yi et al., 2017). Dinoflagellates were more abundant in the copepod diet compared to diatoms, because diatoms were of lower nutritional value than dinoflagellates (Jones and Flynn, 2005). *Isochrysis* and *Mallomonas*, belonging to Chrysophyceae, contain higher levels of unsaturated fatty acid (e.g., DHA, EPA) than many other phytoplanktons, so they have been used as food for laboratory cultured Scallops and zooplankton (Seychelles et al., 2009; Bi et al., 2014; Freites et al., 2016). *Mallomonas*, the high-quality Chrysophyceae, can support high population growth rates and offspring production in zooplankton (Taipale et al., 2019). Since zooplankton are known to preferentially feed on high nutritional quality algae (Taipale et al., 2019), the prey preference of *Polyarthra* can be explained by the nutritional value of the food items.

Both active and passive feeding behavior of zooplankton could be related to the prey size-selectivity (Chen et al., 2021). *Polyarthra* showed preference toward food items with a particle size ranging from 1 to 40 μm (Arndt, 1993). Most Chlorophyceae in the *in situ* diet of *Polyarthra* are well within nano particle size, such as *Chlorella* (5–20 μm)*, Chlamydomonas* (3–10 μm) and *Monoraphidium* (7–20 μm). In addition, *Synura* (2–40 μm), *Chromulina* (3–20 μm), *Mallomonas* (2–16 μm) *Paraphysomonas* (8–17 μm) belonging to Chrysophyta and *Chroomonas* (4–20 μm) belonging to Cryptophyta are also in micro to nano particle size (Hu and Wei, 2006), which are suitable for direct ingestion by *Polyarthra*.

### Feeding strategy responses to trophic status

Copepods can regulate their food intake according to their energy demands when exposed to warming environments. Their feeding preference shift with temperature, with a change from a diatom-dominated diet at 29°C to a metazoan-dominated diet at 35°C (Hu et al., 2018). However, our study indicates that *Polyarthra* adjusted their feeding strategy according to food availability by applying an “opportunistic feeding” strategy. RDA analysis showed that the eukaryotic community of the ambient waters presented a seasonal differentiation pattern and was controlled by temperature and trophic status, which was consistent with the previous studies of zooplankton and phytoplankton communities in Lake Liuye (Liu, 2019; Liang et al., 2020). CCA analysis showed that the eukaryotic community of the *Polyarthra* gut contents also exhibited a distinct pattern of seasonal differentiation, which was consistent with that of the ambient waters. The dietary composition of *Polyarthra* seems to be regulated by environmental factors related to trophic status.

The “opportunistic feeding” strategy is also common for macro-zooplankton as copepod in food-limited conditions (Hu et al., 2020). Dominant species can better their survival by widening the choice of potential food resources (Xu et al., 2020). They are capable to regulate their feeding, by exhibiting a rhythm of herbivorous feeding in midday and carnivorous feeding in morning and night, to better coordinate with other competitors for utilization of food resources (Hu et al., 2020). For example, the genus *Centropages* was more omnivorous-carnivorous and consistently displayed selection for large motile prey when the phytoplankton density was low in the outfall region (Xu et al., 2020). In addition, they can use terrigenous detritus as supplementary food sources when phytoplankton production is limited (Hu et al., 2015). *Polyarthra* mainly grazed on phytoplankton and consumed the animal-derived prey (metazoan detritus or protozoan) as supplementary food sources. In the present study, the spatial–temporal pattern of the eukaryotic community in the *Polyarthra* gut contents varied along an increasing gradient of the trophic status (including TN, TP, Chl-*a*, and SD) in ambient waters (Figure 6B). Moreover, *Polyarthra* mainly feed on Chlorophyceae Trebouxiophyceae, Dinophyceae, Maxillopoda, Chrysophyceae and Synurophyceae when the trophic status was relatively high. To adopt to the relatively lower trophic status, *Polyarthra* had probably shifted from herbivorous feeding to relatively carnivorous (Oligohymenophorea and Spirotrichea) feeding. Our study indicates that *Polyarthra* is omnivorous with preference and responds to the environmental changes by adopting a flexible feeding strategy. This could partly explain why the cosmopolitan rotifers have apparently wide tolerance toward spatial and environmental changes.

### Feeding strategy and ecological function

Carcasses of metazoan will release a continuum of particulate organic matter (POM) and dissolved organic matter (DOM), a complex suite of polymers and molecules, if they are not eaten by predators. These organic matters are further decomposed by bacteria and archaea and converted into inorganic nutrients (e.g., C, N, P, Si and Fe) which can be used by primary producers (Worden et al., 2015). Utilization of DOM and POM by zooplankton might increase the efficiency of the upward trophic transfer of matter and energy flow through secondary consumer under food-limited conditions, and thereby promotes the efficiency of aquatic ecosystem cycling (Hu et al., 2018).

Changes of the diet composition of zooplankton play a key role in the transfer of materials and energy along the food chain (Liu et al., 2006). Phytoplankton density being affected by a feedback mechanism of zooplankton consumer grazing is termed consumer-driven nutrient recycling (Sterner et al., 1995; Trommer et al., 2012). The composition of zooplankton and their excretions can regulate and stimulate the phytoplankton growth and biomass accumulation under N or P limitations (Trommer et al., 2012). Zooplankton feeding on protozoa and metazoan detritus could obtain higher carbon than herbivorous feeding alone. If the POM content of phytoplankton in the water environment is insufficient to satisfy their daily carbon requirements (19 μg), the food intake of protozoa and organic detritus increases (David et al., 2006). Thus, when phytoplankton density was low in Lake Liuye, the high relative abundance of protozoans in the *Polyarthra* gut contents may be related to their carbon requirements. The carnivorous and detritus feeding of zooplankton ensures that secondary consumer can still contribute to the pathways of aquatic carbon flow, through the food chain while under low primary production.

Because feeding strategy of most zooplankton adaptively adjusts with the environment, knowledge of the *in situ* diet composition is crucial for accurately clarifying their ecological function in the aquatic ecosystem, understanding the prey diversity and the ingestion rate (Lin et al., 2018). Our study indicates that HTS is a promising approach that provides us a more comprehensive perspective and more details on the diet composition of microorganism than by microscopic observations. However, to further explore the role and mechanism of zooplankton in biogeochemical cycles, both molecular evidence and microscopy should be combined to investigate the dietary assimilation, release, and physiological turnover rates of C, N and P in zooplanktons under different conditions.

## Conclusion

1. Our HTS data has revealed the dietary information of natural rotifer *Polyarthra* and expanded the knowledge of their *in situ* food resources. A total of 159 OTUs of eukaryotes belonging to phytoplankton, metazoans, protozoans, fungi and other unclassified eukaryotes were detected in *Polyarthra* gut contents.

2. Diet composition indicated that *Polyarthra* is omnivorous with preference and responds to the environmental changes by adopting a flexible feeding strategy. *Polyarthra* mainly grazed on Chlorophyta, which may result from its “opportunistic feeding” strategy. On the contrary, it exhibited a high preference for Chrysophyceae and Synurophyceae.

3 When the concentration of nutrients in the water environment decreased, *Polyarthra* may shift from herbivorous feeding to relatively carnivorous feeding. This could partly explain why the cosmopolitan rotifers have apparently wide tolerance toward spatial and environmental changes.

## Data availability statement

The datasets presented in this study can be found in online repositories. The names of the repository/repositories and accession number (s) can be found at: https://www.ncbi.nlm.nih.gov/, BioProject No. PRJNA611635.

## Author contributions

DL conceived the ideas and led the experiments, data analysis, and paper writing. SL guided paper revising, data analysis, and interpretation. YY were project administrator and supported the research. HL conducted the graphic production. Other authors supported the paper revising and editing. DL, HL, CH, ZY, SS, JD, ML, SL, and YY were critically involved in writing the manuscript. All authors contributed to the article and approved the submitted version.

## Funding

This work received funding from the Guangzhou Municipal Science and Technology Project (No. 202201010592 and 202201010045), the National Natural Science Foundation of China (41673080), and the Fundamental Research Funds for the Central Public Welfare Research Institutes (PM-zx703-202207-267 and PM-zx703-202207-268) for financial support.

## Conflict of interest

The authors declare that the research was conducted in the absence of any commercial or financial relationships that could be construed as a potential conflict of interest.

## Publisher’s note

All claims expressed in this article are solely those of the authors and do not necessarily represent those of their affiliated organizations, or those of the publisher, the editors and the reviewers. Any product that may be evaluated in this article, or claim that may be made by its manufacturer, is not guaranteed or endorsed by the publisher.

## Footnotes

^1^ http://drive5.com/uparse/

^2^ http://rdp.cme.msu.edu/

^3^ http://bioinformatics.psb.ugent.be/webtools/Venn/

^4^ http://www.r-project.org/
