# Supplementary material for: High-throughput sequencing reveals omnivorous and preferential diets of the rotifer Polyarthra in situ
Source: Front Microbiol. 2022 Dec 21;13:1048619. doi: 10.3389/fmicb.2022.1048619 (PMC9810806; doi:10.3389/fmicb.2022.1048619)
Supplement: Supplementary file 1 [file Data_Sheet_1.docx]

Supplementary Material

**High-throughput sequencing reveals omnivorous and preferential diets of the rotifer *Polyarthra* *in situ***

**Table of Contents**

| **Supplementary FIGURE 1** | Page 2 |
| --- | --- |
| **Supplementary Table 1** | Page 3 |
| **Supplementary FIGURE 2** | Page 4 |
| **Supplementary FIGURE 3** | Page 4 |
| **Supplementary FIGURE 4** | Page 5 |
| **Supplementary FIGURE 5** | Page 5 |
| **Supplementary FIGURE 6** | Page 6 |
| **Supplementary Table 2** | Page 6 |
| **Supplementary Table 3** | Page 6 |


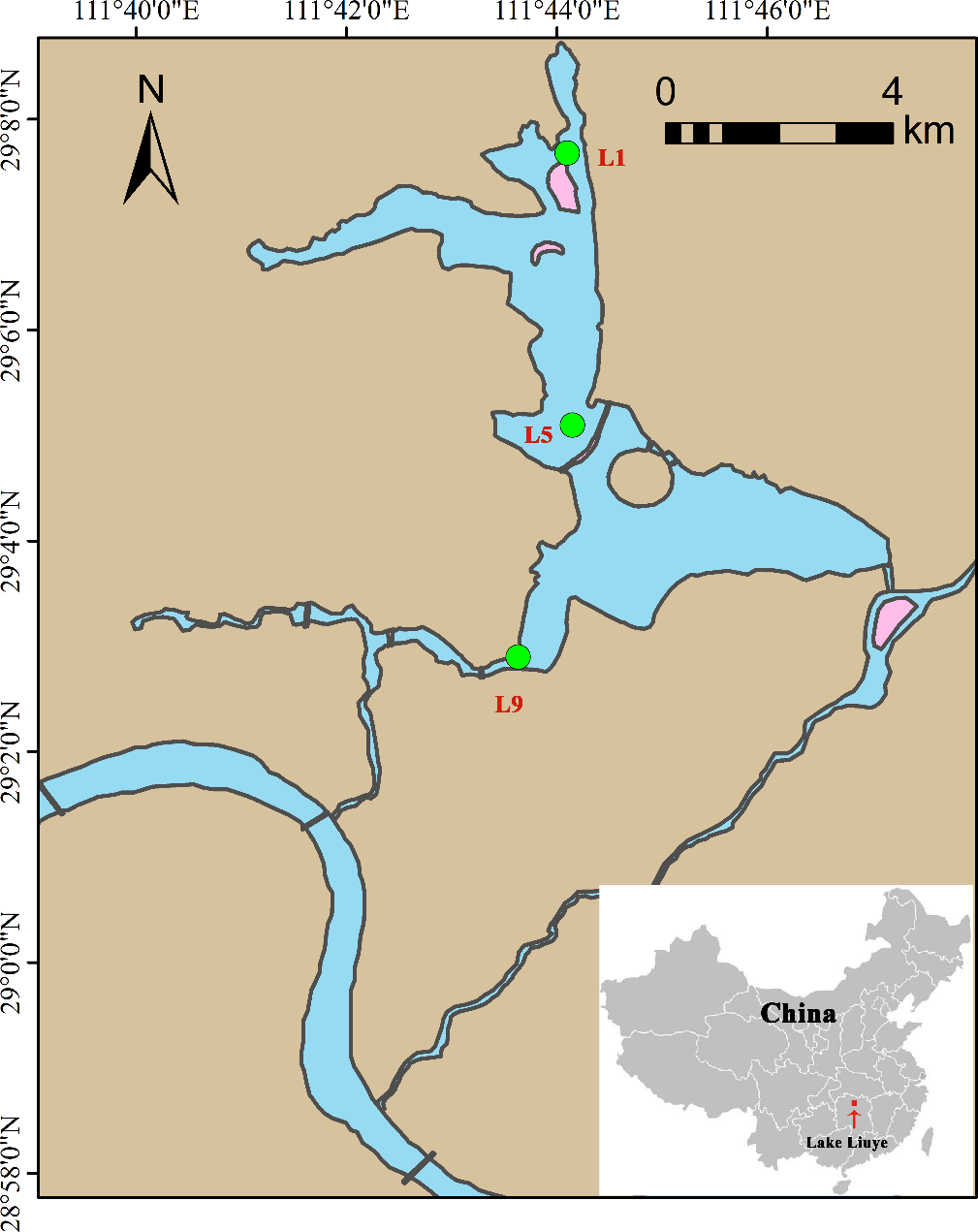


**Supplementary FIGURE 1** Location of the sampling sites in Lake Liuye in the city of Changde, China

**Supplementary Table** 1 Details of sampling localities and the environmental parameters

| Sample ID | Sampling date | Longitude | Latitude | Dep (m) | SD (m) | Temp (℃) | pH | DO (mg/L) | TN (mg/L) | TP (mg/L) | NH_4__N (mg/L) | Chl-*a* (ug/L) | COD (mg/L) |
| --- | --- | --- | --- | --- | --- | --- | --- | --- | --- | --- | --- | --- | --- |
| 12_L1_A | December, 2017 | 111°44'6"E | 29°7'40"N | 3.1 | 0.90 | 7.9 | 7.5 | 10.69 | 3.12 | 0.031 | 0.481 | 2.05 | 3.901 |
| 12_L5_A | December, 2017 | 111°44'6"E | 29°5'6"N | 4.3 | 1.30 | 8.1 | 7.5 | 11.27 | 3.02 | 0.012 | 0.305 | 1.35 | 4.31 |
| 12_L9_A | December, 2017 | 111°43'37"E | 29°2'52"N | 2.8 | 1.10 | 8.1 | 7.6 | 11.08 | 3.34 | 0.016 | 0.675 | 5.22 | 4.31 |
| 3_L1_A | March, 2018 | 111°44'6"E | 29°7'40"N | 2.2 | 0.70 | 14.4 | 8.4 | 11.8 | 5.18 | 0.182 | 0.257 | 14.88 | 4.87 |
| 3_L5_A | March, 2018 | 111°44'6"E | 29°5'6"N | 3.8 | 1.20 | 14.3 | 8.0 | 11.26 | 4.45 | 0.120 | 0.039 | 1.21 | 2.86 |
| 3_L9_A | March, 2018 | 111°43'37"E | 29°2'52"N | 3.1 | 0.90 | 14.6 | 8.1 | 10.75 | 4.73 | 0.120 | 0.048 | 4.14 | 3.31 |
| 6_L1_A | June, 2018 | 111°44'6"E | 29°7'40"N | 1.8 | 0.65 | 28.8 | 8.1 | 5.73 | 9.44 | 0.032 | 0.026 | 14.33 | 10.04 |
| 6_L5_A | June, 2018 | 111°44'6"E | 29°5'6"N | 2.9 | 0.45 | 27.8 | 8.0 | 5.31 | 9.35 | 0.048 | 0.028 | 7.85 | 9.98 |
| 6_L9_A | June, 2018 | 111°43'37"E | 29°2'52"N | 3 | 0.60 | 29.3 | 8.7 | 7.78 | 10.31 | 0.132 | 0.121 | 18.53 | 11.27 |
| 9_L1_A | September, 2018 | 111°44'6"E | 29°7'40"N | 2.9 | 0.60 | 25.2 | 7.8 | 6.24 | 11.22 | 0.038 | 0.011 | 2.90 | 8.37 |
| 9_L5_A | September, 2018 | 111°44'6"E | 29°5'6"N | 4.1 | 0.72 | 26.1 | 7.7 | 6.3 | 8.18 | 0.022 | 0.020 | 3.09 | 8.48 |
| 9_L9_A | September, 2018 | 111°43'37"E | 29°2'52"N | 3.9 | 0.80 | 26.9 | 7.9 | 6.13 | 11.13 | 0.024 | 0.003 | 4.72 | 8.28 |

data from Liang et al., 2020


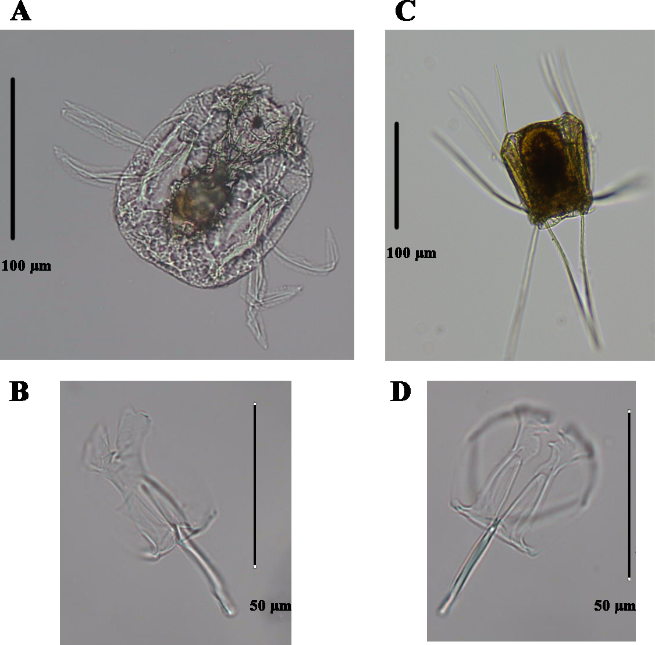


**Supplementary FIGURE 2** Morphologic photographs: *Polyarthra vulgaris* (A); the trophi of *P. vulgaris* (B); *P. dolichoptera* (C); the trophi of *P. dolichoptera* (D). (data from Liang et al., 2022)


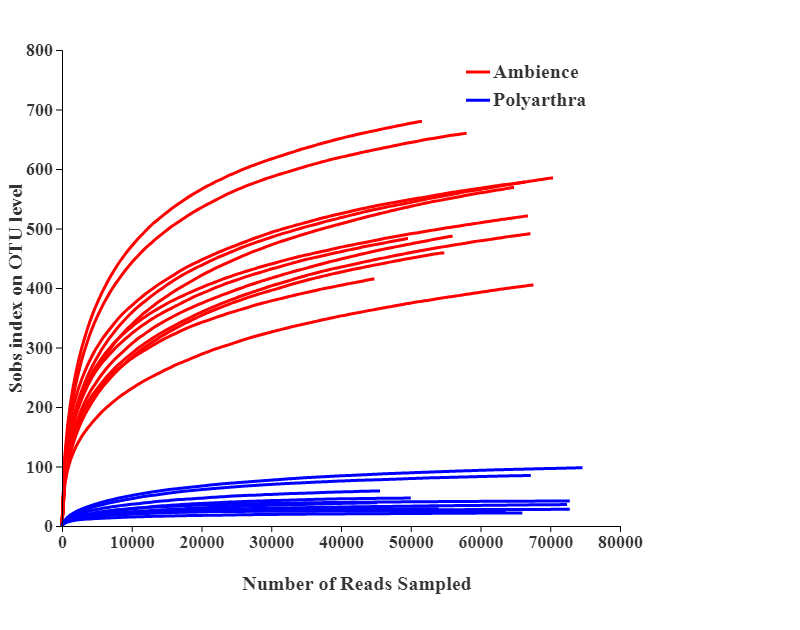


**Supplementary FIGURE 3** Rarefaction curves of all samples (red line, ambient water samples; blue line, *Polyarthra* samples)


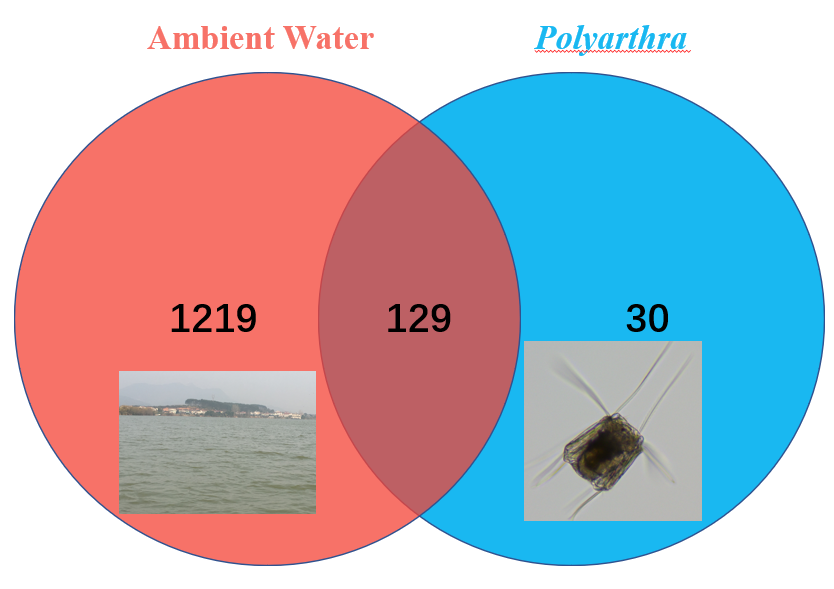


**Supplementary FIGURE 4** Venn diagram showing the numbers of unique and shared OTUs between the ambient waters and the *Polyarthra* gut contents samples


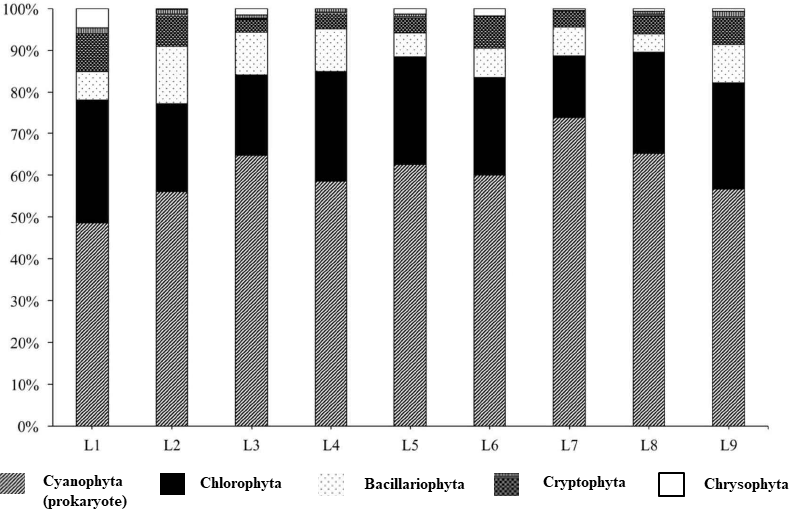


**Supplementary FIGURE 5** Average relative abundance of dominant phytoplanktons (phylum level) in Lake Liuye during 2018, based on the morphological approach. (data from Liu, 2019)


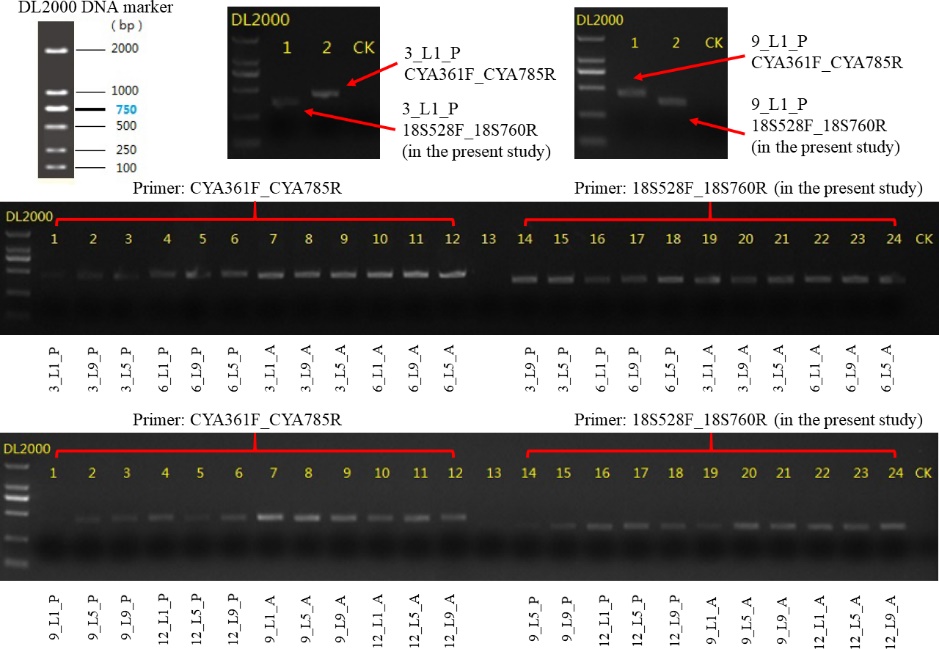


**Supplementary FIGURE 6** 18S rDNA PCR reactions of the 24 samples. CK: negative control reactions (double distilled water). Since the target bands size of PCR product were correct and the color is moderate, subsequent experiments can be carried out.

**Supplementary Table 2** Correlation coefficient between environmental factors and the first two ordination axes of the eukaryotic community in the ambient waters

|  | RDA1 | RDA2 | R^2^ |
| --- | --- | --- | --- |
| Dep | -0.4924 | -0.8704** | 0.1887 |
| SD | -0.9283** | -0.3717 | 0.4343 |
| Temp | 0.9961** | 0.0877 | 0.8019 |
| TP | -0.1223 | -0.9925** | 0.2029 |
| NH_4_-N | -0.8721** | 0.4894 | 0.8564 |
| Chl-*a* | 0.6369* | 0.771** | 0.281 |

*：*p* < 0.05；**：*p* < 0.01；

**Supplementary Table 3** Correlation coefficient between environmental factors and the first two ordination axes of the eukaryotic community in the *Polyarthra* gut contents

|  | CCA1 | CCA2 | R^2^ |
| --- | --- | --- | --- |
| Dep | 0.2975 | 0.9547** | 0.5252 |
| SD | -0.9614** | -0.2751 | 0.4632 |
| TN | 0.9693** | 0.2458 | 0.8663 |
| TP | 0.1917 | -0.9814** | 0.3399 |
| Chl-*a* | 0.397 | -0.9178** | 0.5147 |

*：*p* < 0.05；**：*p* < 0.01

Reference

Liang, D., Wang, Q., Wei, N., Tang, C., Sun, X., & Yang, Y. (2020). Biological indicators of ecological quality in typical urban river-lake ecosystems: The planktonic rotifer community and its response to environmental factors. *Ecological Indicators, 112*, 106127. https://doi.org/10.1016/j.ecolind.2020.106127

Liang, D., McManus, G. B., Wang, Q., Sun, X., Liu, Z., Lin, S., & Yang, Y. (2022). Genetic differentiation and phylogeography of rotifer *Polyarthra dolichoptera* and *P. vulgaris* populations between Southeastern China and eastern North America: High intercontinental differences. *Ecology and evolution*, 12(5), e8912. https://doi.org/10.1002/ece3.8912
